# Haptic Devices Based on Real-Time Dynamic Models of Multibody Systems

**DOI:** 10.3390/s21144794

**Published:** 2021-07-14

**Authors:** Nicolas Docquier, Sébastien Timmermans, Paul Fisette

**Affiliations:** Mechatronic, Electrical Energy, and Dynamic Systems (MEED), Institute of Mechanics, Materials and Civil Engineering (iMMC), Université Catholique de Louvain, 1348 Louvain-la-Neuve, Belgium; sebastien.timmermans@uclouvain.be (S.T.); paul.fisette@uclouvain.be (P.F.)

**Keywords:** multibody dynamics, symbolic generation, real-time computation, human-in-the-loop, haptic devices

## Abstract

Multibody modeling of mechanical systems can be applied to various applications. Human-in-the-loop interfaces represent a growing research field, for which increasingly more devices include a dynamic multibody model to emulate the system physics in real-time. In this scope, reliable and highly dynamic sensors, to both validate those models and to measure in real-time the physical system behavior, have become crucial. In this paper, a multibody modeling approach in relative coordinates is proposed, based on symbolic equations of the physical system. The model is running in a ROS environment, which interacts with sensors and actuators. Two real-time applications with haptic feedback are presented: a piano key and a car simulator. In the present work, several sensors are used to characterize and validate the multibody model, but also to measure the system kinematics and dynamics within the human-in-the-loop process, and to ultimately validate the haptic device behavior. Experimental results for both developed devices confirm the interest of an embedded multibody model to enhance the haptic feedback performances. Besides, model parameters variations during the experiments illustrate the infinite possibilities that such model-based configurable haptic devices can offer.

## 1. Introduction

Multibody formalisms, which appeared in the seventies, were intended to produce the equations of motion of so-called multibody systems (MBS) of any size and of any kind, with the aim of studying their motion. For several decades, multibody models were mainly used to study mechanical systems with a purely predictive (development of a new device) or corrective (improvement of an existing device) target. The “real-time computation” character was completely absent for obvious limitations related to the performance of computers at the time.

The massive and amazing arrival of increasingly more powerful processors and of faster and larger memories (RAM) encouraged research teams to produce very compact multibody formalisms (e.g., based on so-called recursive techniques or Order-N methods), to exploit programming techniques (such as symbolic generation) and to take maximum advantage of state-of-the-art computer architectures (vector or parallel processors).

All this contributed to exploring the possibilities of using multibody models in the context of real-time computing. Already at the beginning of the nineties for instance, the inverse dynamic computation of robot actuator torques was carried out to enrich their internal controllers with a feedforward component (e.g., predicted-torque control). Computational algorithms in haptic rendering may consider various approaches [1].

Among demanding applications (in terms of real-time computation point of view), one finds a series of multibody systems (vehicle simulator, remotely-actuated surgical robot, etc.) whose haptic feedback (i.e., the force or torque to be sent back to the human) should reproduce, as accurately as possible, the dynamics of the system with which it is in interaction. Among the ingredients required to develop such devices one finds, beside the real-time multibody model, a series of sensors (at the human–machine and machine–machine interfaces), powerful computer processors, and an operating meta-system. The latter must play the role of a real conductor responsible for synchronizing and interfacing the involved hardware and software components.

This work presents the developments realized in this sense by the UCLouvain multibody team, who decided to make the most to couple the symbolic multibody models (issuing from their symbolic ROBOTRAN (www.robotran.eu, accessed on 13 July 2021) software) within the ROS (www.ros.org, accessed on 13 July 2021) architecture (Robot Operating System). The latter is used to interface the models with a set of well-chosen sensors pursuing two objectives: (i) first to validate the multibody models with respect to experimental physics and (ii) second to handle specific haptic devices whose underlying physical system is dynamic in nature.

Two very distinct applications are presented to illustrate the approach. The first one—of research type—concerns the development of a digital piano keyboard with haptic feedback, based on the real-time multibody model of the grand piano action. The second application—rather educational—concerns a car simulator whose 3D multibody model allows to feedback pertinent sensory information, namely, the steering torque and a 3D visualization with realistic visual clues. The model can be tuned in real-time by instantaneously modifying the suspension settings but also the contact characteristics of the tire/ground forces.

In all cases, the haptic specifications and requirements of the envisaged applications have guided our choices for (i) the electromechanical design itself, (ii) the level of refinement and computational efficiency of the multibody model, (iii) the type and conditioning of the sensors, and (iv) the way of interfacing the whole through a suitable meta-architecture (ROS). The results confirm the advantages and assets of the symbolic multibody approach and the interest of embedding such models in haptic devices to best capture highly dynamic effects (a technique that was not conceivable with the technologies of the past in a context of haptic feedback).

The paper is organized as follows. After a state-of-the-art on multibody formalisms and haptic devices (Section 2), the multibody approach and its symbolic implementation are treated in Section 3. Section 4 addresses the different aspects of the haptic problem, still focusing on multibody applications. Section 5 illustrates the approach proposed for the two above-mentioned applications and, more succinctly and illustratively, for other systems developed in our laboratory. A concluding section closes this work, pointing out the interesting perspectives of this research. Having a immense potential, haptics technology is still in its beginning stage [2].

## 2. State-of-the-Art

### 2.1. Multibody Formalisms

**Figure 1:**
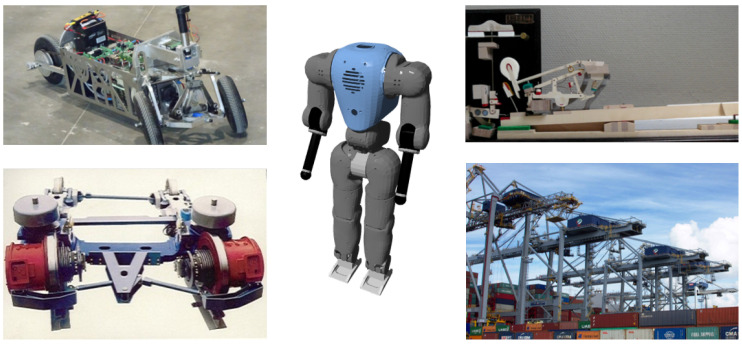
Multibody applications range from small piano action, through road and railway vehicles, to huge port cranes (UCLouvain).

Multibody dynamics, a scientific discipline that emerged in the early seventies (see, e.g., in [3,4]), is concerned with the kinematic and dynamic study of “polyarticulated” mechanical systems (referred to as MultiBody Systems, or MBS in short) such as transmission mechanisms, humanoid robots, road and railway vehicles, human body, cranes, etc. (Figure 1).

Beside experimental investigations, the analysis of the motion of these MBS requires their mathematical modeling, based on the fundamental laws of mechanics, of which the Newton equations (for body translations) and Euler equations (for body rotations) are common starting points, in the same way as the Lagrange equations or the virtual work and virtual power principles.

To obtain the equations describing the motion of MBS, several choices of variables (referred to as generalized coordinates *q*) are possible. Some schools have favored absolute or nodal coordinates (denoting the configuration of each body of the MBS with respect to an inertial frame) [4,5,6], others preferred using relative coordinates (representing the configuration of each body with respect to another body of the MBS) [7,8] or natural coordinates (representing the absolute configuration of specific material points on each body of the MBS) [9].

Concerning the bodies themselves, the formalisms are distinguished according to whether they consider them rigid or flexible, in which case deformation equations are superimposed to the equations of the overall motion of the MBS (see. e.g., in [6,8]).

Regardless of the formalism used, the complexity of real applications is such that the automatic generation of their multibody models on a computer has quickly become essential. Indeed, it allows obtaining reliable and sufficiently generic models to deal with the numerous MBS families (see above). A distinction is made between the numerical approach (e.g., Adams-MSC or Samcef-Mecano software) and the so-called symbolic approach (e.g., Neweul-M2, Maplesoft, and ROBOTRAN software) which still presently cohabit among the available multibody computer programs.

The first approach requires reconstructing the model through a series of numerical subroutines at each step of the computation (e.g., integration time step). This reconstruction inevitably drains a series of useless computations linked to the numerous zeros of multibody models (inherent to the data or to the tree-like morphology of the physical system). Symbolic multibody programs avoid this algorithmic reconstruction: the whole dynamic model fits in a single file. Above all, symbolic engines can eliminate unnecessary operations like multiplication by zero, condensation of trigonometric formulas or even deletion of complete unnecessary equations provided by generic multibody formalisms [10]. Their superiority in terms of FLOPS is established and leads to much lower computation times, favorable to the real-time performances required here. In sum, symbolic multibody-dedicated programs allow to produce compact equations leading to very efficient and portable models, written in the desired language (e.g., C, Python, Matlab).

Multibody dynamics then opened up to other physics disciplines (e.g., hydraulics, pneumatics, mechatronics, and granular media) and to specific analysis methods (control, optimization, and HIL real-time applications), the challenge being essentially to ensure a numerically robust and computationally efficient coupling between the mathematical models of the disciplines involved (see, e.g., in [11]). The scientific literature of the last twenty years is full of developments in this field of multibody-multiphysics coupling, most often guided by a specific type of application (see, e.g., in [12]).

For systems requiring real-time (or even faster than real-time) computation of their multibody model, as in the case of haptic devices whose internal kinematics or dynamics cannot be disregarded, specific developments have been carried out by the scientific community. They allow, among others, to achieve feedforward computed-torque control of walking robots, to increase the precision of surgical tools or to improve the haptic feedback of driving simulators.

In this context, one of the current challenges concerns the inclusion in haptic feedback devices of dynamic models that are (i) sufficiently representative of the system (number of bodies, joints, nature of internal forces, etc.), (ii) accurate (correct identification of parameters and force laws), and (iii) efficient (i.e., faster than real-time). All this cannot be achieved without a strong interaction between the virtual model, the real device and the external world, through the use of both virtual and real sensors, in order to claim high-level haptic performances.

### 2.2. Hardware Implementation

On one hand, validation of a multibody model thanks to specific sensors is widespread and ranges from many applications with various sensors, for example, a railway track geometry measurement based on IMUs, cameras, and encoders [13]; model estimation of a vehicle suspension system with strain gauges [14]; gait analysis through IMUs compared to optical motion capture [15]; validation of a axial piston pump model via force and pressure sensors [16]; and acceleration and force piezoelectric sensors to test a wind turbine flexible multibody model [17].

On the other hand, a growing number of researches has been carried out to use sensors in real-time to measure the physical system behavior. This information is crucial to properly feed the multibody model.

For example, in 2016, Torres-Moreno et. al focused on an online dynamic estimation of the four-bar mechanism [18]. The kinematics of the system is retrieved through IMUs measurements and optical encoders. They proposed an extended Kalman filter approach that allows to deal with constrained multibody system. The estimators are run in real-time and experiments show good agreements with simulation results.

Running a multibody model in real-time requires sufficient computational power on the considered hardware. While it is possible to perform the online computation on a classic computer with *Intel* processor and *Windows* OS [19], it may not be the most robust solution. Besides, if one aims to perform an embedded simulation, the hardware is often limited. In this regard, some researches have achieved to run multibody models on other kinds of platforms, such as FPGA [20] or ARM-based systems [21]. In particular, the above-mentioned symbolic approach has been proved to be a good candidate to achieve real-time computation without having to resort to simplifications within the model [22].

Concerning the software implementation, new tools have been provided by the scientific community to combine integrated devices and simulators, mainly in the robotic field. The so-called *middleware* platforms are able to manage the communications between the different interfaces and facilitate the code modularity and reuse. Rivera presented a recent summary of the middleware platforms in [23], while using a Gazebo/ROS-based environments for his application. The ROS environment is now widely used for real-time robotics, for example, in [24] to represent a complex robot real-time dynamic simulator.

In the recent past, our multibody software ROBOTRAN has been coupled with the YARP interface for humanoid applications [25], combining the real robot with the simulated one. Force–torque sensors and IMUs have been used in reality and successfully matched with their simulation equivalent.

Despite all these researches, the real-time multibody models are still rarely embedded in haptic devices, the goal being to improve the feedback for “rigid–rigid interaction” type applications, which only represent one of the multiple components of the Haptic rendering discipline [26]. Haptic devices often consider simplified dynamics to synthesize the haptic feedback, for example in the REPLALINK prototype [27] or in a piano action model [28] (see also Section 5.1). Advanced haptic devices embedding a full multibody model begin to appear in the literature [29,30], exhibiting promising prospects thanks to the conjoint evolution of modeling, computational and technological tools.

## 3. Multibody Formalism

Among the modeling options presented in Section 2.1, we have opted for a relative coordinate formalism (Section 3.1) and a symbolic implementation of multibody equations (Section 3.2). This combination offers the advantage of producing models that can be easily interfaced, either with other disciplines (in a multiphysics perspective) or within a middelware platform (to couple devices and software in a real-time process).

### 3.1. Modeling MBS Using Relative Coordinates

**Figure 2:**
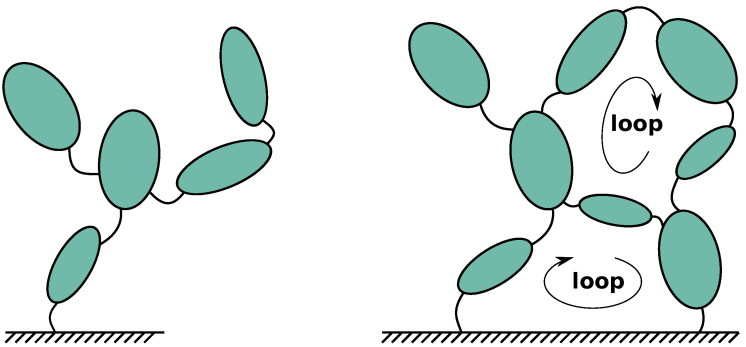
Tree-like (**left**) and closed-loop (**right**) MBS topologies.

When choosing relative coordinates, a MBS can be built on the basis of its topology characterized by the successive bodies and joints, as shown in Figure 2, on the left for a tree-like MBS (such as a serial robot) and on the right for a MBS containing kinematic loops (such as a multi-link car suspension). The relative angular (*rad*) or linear (*m*) displacements within the joints are called the generalized coordinates, *q*, of the MBS (also called *joint coordinates*). These are independent for a tree-like MBS but not for a closed-loop one, each loop of bodies leading to geometrical constraints, denoted as *h*(*q*) = 0, between the joint coordinates present in the loop. The distinction between tree-like and closed-loop MBS is essential because it is at the root of the relative coordinate formalisms as synthetically explained below.

Tree-Like MBS Modeling

Basically (see in [8] for more details), in the case of a tree-like MBS containing *n* joint coordinates, these confer *n* motion degrees of freedom (DOF) to the system, leading to a set of *n* equations of motion.

Various techniques can be envisaged to obtain them (d’Alembert principle, Lagrange equations, Newton–Euler equations recursively computed on the topology [8]). In any case, one ends up with a form equivalent to the following one (“.” stands for $\frac{d}{dt}$ and “..” for $\frac{d^{2}}{dt^{2}}$):

(1) $$M\left(q\right)\;\ddot{q}+c\left(q,\dot{q},frc,trq\right)=Q\left(q,\dot{q}\right),$$

in which $\dot{q}$ and $\ddot{q}$, respectively, stand for the generalized relative velocities and accelerations; M(q) represents the system mass matrix; $c(q,\dot{q})$ gather the centripetal, Coriolis, gyroscopic terms as well as the external forces $frc(q,\dot{q})$ and torques $trq(q,\dot{q}$); and $Q(q,\dot{q})$ denotes the efforts within the joints (actuators or passive elements).

Equation (1) can be straightforwardly solved using linear algebra to obtain the relative accelerations $\ddot{q}$: this refers to *Direct Dynamics*. This computation has a $O(n^{3})$ computational complexity due to the factorization of the mass matrix. Among other things, direct dynamics allows, via the time integration of the accelerations, to determine the time history of the positions q(t) and velocities $\dot{q}\left(t\right)$ and thus to predict the motion of the MBS subjected to internal or external forces, starting from initial conditions q(0), $\dot{q}\left(0\right)$.

As an illustration, direct dynamics is widely used for road and railway vehicles to predict, analyze, or optimize their dynamic performances. Direct dynamics is also used in various driving simulators to make haptic feedback more accurate and realistic (see Section 5.2).

*Inverse Dynamics* is another way of using Equation (1) for applications where the MBS trajectory is known (q(t), $\dot{q}\left(t\right)$, and $\ddot{q}\left(t\right)$) and for which the unknowns are now the joint forces and torques Q(t) and not the accelerations anymore. Equation (1) can then be written as follows:

(2) $$Q\left(q,\dot{q}\right)=\phi \left(q,\dot{q},\ddot{q},frc,trq\right)$$

with

$$\phi =M\left(q\right)\;\ddot{q}+c\left(q,\dot{q},frc,trq\right)$$

As the explicit computation of the mass matrix *M* is not necessary anymore to compute *Q* via (2), ϕ can be generated without explicitly computing it, which leads to an efficient O(n) computational complexity of the formalism.

For instance, inverse dynamics is useful to compute the actuator torques of robots for evaluating a feedforward component in their motion controller.

Closed-Loop MBS Modeling

A multibody system can be subject to different types of constraints between its generalized coordinates, *q*, which can be holonomic (i.e., algebraic, like assembly constraints), non-holonomic (expressed at the level of velocities, like for a slip-free rolling), and/or rheonomic (when they depend explicitly on time *t*). In the present context of haptic devices modeling, we will limit ourselves to the holonomic case, focusing on kinematic loop constraints; this does not exclude the case of other algebraic constraints that would be treated in the same way by the following formalism.

When dealing with closed-loop MBS (as depicted on the right of Figure 2), *m* loop constraints between the generalized coordinates *q*, denoted h(q)=0, must be solved in parallel to the equations of motion (1) or (2), in which an additional term is required to take the constraint forces and torques into account, according to the well-established Lagrange multipliers technique. For the direct dynamics, the final form reads

(3) $$\begin{array}{ccc} M\left(q\right)\;\ddot{q}+c\left(q,\dot{q},frc,trq\right) & = & Q\left(q,\dot{q}\right)+J^{t}\left(q\right)\;\lambda \\ h\left(q\right)=0\;\;;\;\;\dot{h}\left(q,\dot{q}\right)=J\left(q\right)\;\dot{q} & = & 0\;\;;\;\;\ddot{h}\left(q,\dot{q},\ddot{q}\right)=0 \end{array}$$

And in a similar way, for the inverse dynamics the equations becomes

(4) $$\begin{array}{ccc} Q(q,\dot{q}) & = & \phi \left(q,\dot{q},\ddot{q},frc,trq\right)-J^{t}\left(q\right)\;\lambda \\ h(q) & = & 0\;\;;\;\;\dot{h}\left(q,\dot{q}\right)=J\left(q\right)\;\dot{q}=0\;\;;\;\;\ddot{h}\left(q,\dot{q},\ddot{q}\right)=0 \end{array}$$

in which $J\left(q\right)≜\frac{\partial h}{\partial q^{t}}$ denotes the constraint Jacobian matrix and λ are the Lagrange multipliers associated with the constraints h(q). In both cases, the number of DOF equals n−m, that is the number of generalized coordinates *q* minus the number of independent constraints *h* (The case of redundant constraints is treated within Robotran, but only for “classical” multibody simulations, and not for the proposed haptic coupling. As there were no redundant constraints for the applications treated in this paper, this topic has not been discussed.).

**Figure 3:**
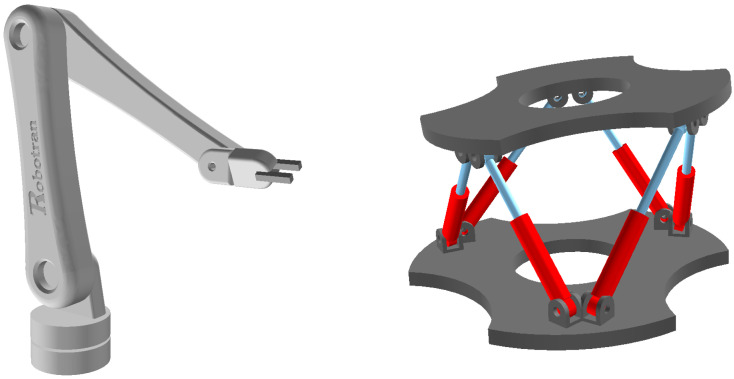
Serial (**left**) versus parallel (**right**) manipulators.

Let us illustrate closed-loop morphologies in the field of robotics. An anthropomorphic serial robot with 6 actuated joints (Figure 3 (left)) possesses 6 DOFs. On the other hand, a parallel structure such as the Stewart platform (Figure 3 (right)) has 18 joints embedded into multiple loops of bodies, but its upper-plate only possesses 6 DOFs and its motion is ensured by 6 actuators forming the legs of the platform.

Equations (3) or (4) form a DAE system, as they are made of differential and algebraic equations, whose direct solving is not trivial. A practical and elegant way to solve them is to transform them into ordinary differential equations (ODE), using the so-called *Coordinate Partitioning Method* briefly presented below (see in [4,8] for more details).

The starting point of the method refers to the number of degrees of freedom (n−m) of the system, to which (n−m) generalized coordinates can match. They are called *independent* coordinates, denoted *u*, while the others, denoted *v*, are *dependent*. Therefore, we can partition the column vector *q* as follows:

(5) $$q=\left(\begin{array}{c} u\\ v \end{array}\right)$$

By applying this partitioning to all the matrices and vectors appearing in Equation (3) or (4), and by expressing the dependent coordinates via the solving of the constraints at position, velocity and acceleration levels,

(6) $$\begin{array}{ccc} h(q)=0 & \Rightarrow & v=f(u),\\ \dot{h}\left(q,\dot{q}\right)=0 & \Rightarrow & \dot{v}=g\left(q,\dot{u}\right),\\ \ddot{h}\left(q,\dot{q},\ddot{q}\right)=0 & \Rightarrow & \ddot{v}=h\left(q,\dot{q},\ddot{u}\right), \end{array}$$

it is possible, after a few matrix manipulations, to end up with a reduced set of equations of motion in terms of the independent coordinates *u*:

(7) $$\begin{array}{ccc} M_{r}\left(u\right),\ddot{u}+c_{r}\left(u,\dot{u},frc,trq\right) & = & Q_{r}\left(u,\dot{u}\right) \end{array}$$

(8) $$\begin{array}{ccc} Q_{r}\left(u,\dot{u}\right) & = & \phi_{r}\left(u,\dot{u},\ddot{u}\right), \end{array}$$

for the direct and inverse dynamics, respectively.

The coordinate partitioning technique amounts to projecting the equations of motion in the MBS motion manifold, with the advantage of getting rid rigorously of the algebraic constraint equations. Therefore, one ends up with a ODE system of minimum size (n-m) which exactly corresponds to the number of DOF of the MBS (ex. n−m=6 for the Stewart platform of Figure 3).

It is worth mentioning that the resolution of the constraints (6) can be very accurate using an iterative (for *h*) or an algebraic (for $\dot{h}$ and $\ddot{h}$) algorithm, at each evaluation of the dynamic equations.

Driven Motion

When dealing with MBS in which the time evolution of some of the generalized coordinates *u* is fully imposed (such as for instance in robotics when motion control is achieved), the corresponding joints are qualified as *driven*. The time history of the associated coordinates is thus a prescribed function of time *t*. To take them into account in the above formalism, we can apply a second partitioning, but on *u* in the present case, that is,

(9) $$u=\left(\begin{array}{c} u_{u}\\ u_{d} \end{array}\right),$$

in which $u_{u}$ represents the new subset of independent coordinates and $u_{d}$ stands for the driven coordinates viewed, from the MBS point of view, as known functions of time at position, velocity, and acceleration levels:

(10) $$u_{d}=f\left(t\right)\;\;;\;\;{\dot{u}}_{d}=\frac{du_{d}}{dt}\;\;;\;\;{\ddot{u}}_{d}=\frac{d^{2}u_{d}}{dt^{2}}$$

Note that driven variables can also be used to deal with rheonomic constraints. Indeed, they allow to describe an explicit time dependence of any function in which they appear, as in an algebraic constraint for example.

Introducing the partitioning (9) into the equations of motion (7) gives

(11) $$\left(\begin{array}{cc} M_{r}^{uu} & M_{r}^{ud}\\ M_{r}^{du} & M_{r}^{dd} \end{array}\right)\left(\begin{array}{c} {\ddot{u}}_{u}\\ {\ddot{u}}_{d} \end{array}\right)+\left(\begin{array}{c} c_{r}^{u}\\ c_{r}^{d}\; \end{array}\right)=\left(\begin{array}{c} Q_{r}^{u}\\ Q_{r}^{d} \end{array}\right)$$

The first line of (11) can be solved with respect to ${\ddot{u}}_{u}$ and represents the final form of a *direct dynamics* problem (to be time integrated with respect to the $u_{u}$ variables for instance):

(12) $$M_{r}^{uu}{\ddot{u}}_{u}+c_{r}^{u}=Q_{r}^{u}-M_{r}^{ud}{\ddot{u}}_{d}$$

Let us note that this equation can also be formulated in an inverse dynamics form, in a similar way to Equation (8).

The second line of (11) can be put aside if we are only interested in analysing the motion of the MBS Equations (12). However, those equations can be very useful to compute efforts ($Q_{r}^{d}$) associated with the driven coordinates (ex. a motor torque, a force in an mechanical assembly or a reaction force in a bearing):

(13) $$Q_{r}^{d}=M_{r}^{du}{\ddot{u}}_{u}+M_{r}^{dd}{\ddot{u}}_{d}+c_{r}^{d}$$

In a certain manner, this equation represents an inverse dynamics computation carried out in parallel with—or as a postprocess of—the direct dynamics (12).

Implementation in a Haptic Context

**Figure 4:**
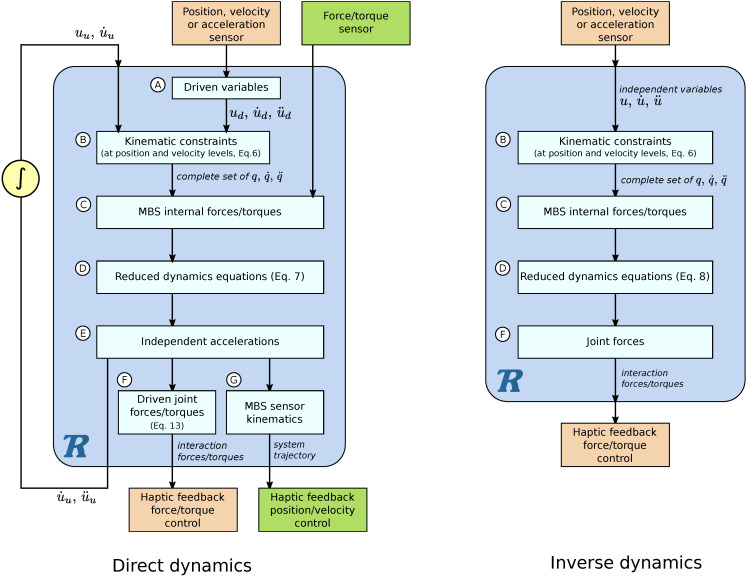
Flow diagram of direct and inverse dynamics in view of coupling them with haptic devices.

The multibody computation workflow varies slightly whether direct dynamics or inverse dynamics are considered (Figure 4). For direct dynamics simulations, a numerical integrator computes the time evolution of the independent variables. Trajectory sensors (i.e., position, velocity, or acceleration sensors) define the motion of *driven joints* (Step A), such as the angular position of a steering wheel for a driver simulator or the displacement of a piano key (see Section 5). When the trajectories of all independent joints are measured, the inverse dynamics formulation is used. Filtering, differentiating or integrating the sensor signal may be necessary.

The algebraic constraints (6) of closed-loop MBS are used to compute dependent positions and velocities (step B). Once the configuration of the system is fully defined, external and internal forces are computed, for instance, the tyre/ground interaction for a car or the contact interaction between the parts of a piano action (step C). At this stage, inputs coming from force/torque sensors can be considered in the dynamics. The dynamic Equations (3) or (4) are computed and, for closed-loop systems, the coordinate partitioning technique is applied (step D) to get the reduced set of Equation (7). In the case of direct dynamics, a linear system of equations must be solved (step E) to compute the independent acceleration that will be handled by the time integrator. For the inverse dynamics, joint forces/torques are derived directly from the dynamics (step F) using (8).

Forces or torques associated with driven joints are computed (step F) using (13), which enables computing a feedback that takes the dynamics into account, like the wheel steering torque on the driver or the reaction of the piano key on the musician finger. In addition, any kinematic information of the system, such as the position or velocity of any point, can also be computed (step G) and be used for the system feedback using so-called *multibody sensors*.

### 3.2. Symbolic Multibody Model

**Figure 5:**
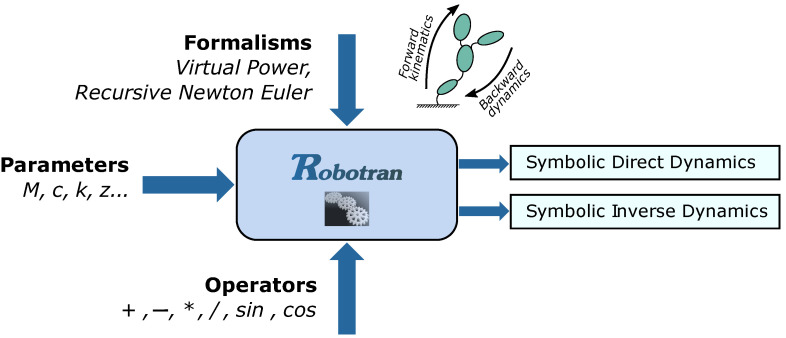
Symbolic Generation Process.

The aim of the symbolic generation of multibody models is to take advantage of both the genericity inherent to a numerical generation of multibody equations and of the simplification potentialities of their manual writing. So-called *symbolic* multibody programs manipulate arithmetical operators (+, −, *, /) and alphanumeric strings representing the data (*m*, *c*, *K*, etc., see Figure 5) to generate analytical equations in C, Python, or MATLAB, making the most of arithmetical and trigonometric simplifications. For a generic 3D MBS, the symbolic generation is performed only once (in less than one second with ROBOTRAN for MBS containing a hundred DOF) prior to any numerical analysis.

The computational superiority of the symbolic vs. the numerical approach is particularly attractive since it gives the opportunity to

- significantly speed up the computation of MBS models; this represents a very valuable asset for applications requiring real-time computing, such as those targeted here, and
- easily couple multibody models—being encapsulated in symbolic files—to other disciplines (such as optimization, control, electromechanical dimensioning) at software but also at hardware levels.

These two advantages are widely exploited in the applications underlying this work as detailed in the next sections.

ROBOTRAN Symbolic Generation

This section summarizes the key elements of the symbolic generation underlying the multibody software ROBOTRAN, the details of which can be found in reference [10], dedicated to this precise topic. There do obviously exist commercial general-purpose symbolic packages: why do we not use these to generate multibody equations?

The first reason relates to the amount of computer memory required to generate large symbolic models (up to 400 DOFs in our case). The combined use of dynamic memory allocation (via the use of C-pointers) and of the linked lists technique, allows us to finely control the ROBOTRAN memory requirement during the generation process.

The second reason concerns the possible simplifications of mathematical alphanumeric expressions. Although the simplification capabilities of commercial packages are extremely powerful, the symbolic condensation of multibody equations relies on a set of rules and tricks which are specific to MBS, namely, the simplification of complex trigonometric formulae and kinematic expressions, the pure and lasting elimination of unnecessary recursive equations and the parallelization of the latter, among others.

Arithmetic and Trigonometric Simplifications

To simplify multibody expressions containing unnecessary null data or similar terms which cancel each other, any encountered expression is recursively reorganized by ROBOTRAN on the basis of a so-called *multibody priority rule* (e.g., a force symbol has a lesser priority than a mass symbol but has a higher priority than a generalized coordinate) as illustrated in the following priority “<” relationship:

(14) $$m^{i}<d^{ij}<F_{ext}^{i}<q^{i}<sin,cos<b*c<\left(a-d\right)$$

in which we have intentionally classified expressions with respect to their physical meaning (ex.: mass, length, force, generalized coordinates, sine and cosine, etc.) in addition to their arithmetical nature.

For purely illustrative purposes, here are some excerpts of the symbolic generation of the inverse dynamics of a mechanism (articulation 2). qk, qdk, and qddk, respectively, represent the generalized coordinate, velocity and acceleration associated with joint *k*, mi, and bij are, respectively, masses and barycentric vectors. Dij and Ljk are geometric vectors.

Pre-computations:

S1=sin(q1);S2=sin(q2);C1=cos(q1);S3=sin(q3);

q1p2=q1+q2;q2p3=q2+q3;q1p2p3=q1p2+q3;

S1p2=sin(q1p2);S2p3=sin(q2p3);S1p2p3=sin(q1p2p3);

mb2=m2+m3;b11=m1*L11;b21=m2*L12+m3*D13;b31=m3*L13;b32=m3*L23;

Inverse dynamics (excerpt):

Q(2)=b11*qdd1*C1…−b31*qd1*qd1*S1p2p3…−2*b21*qd1*qd2*S1p2…−mb2*g.

When dealing with a given symbolic expression (whatever its length and complexity), the ROBOTRAN symbolic engine *recursively* refers to the above priority rule (14) to ensure that the final form of any expression will be *purged* of zero parameters and of consecutive identical terms—or sub-expressions—with opposite signs.

As regards trigonometric expressions, which can be very numerous in MBS due to the 3D rotations, an internal process is activated to drastically simplify them, as illustrated by the following example (in which *C* and *S* stand for the sine and cosine functions, respectively, and Sij denotes the sine of the sum of the generalized coordinates $q^{i}$ and $q^{j}$).

C2*C4*C56*C56*S8+C2*C4*S56*S56*S8+C2*S4*S56*C8

+S2*C4*S56*C8−S2*S4*C56*C56*S8−S2*S4*S56*S56*S8

is automatically simplified on-line by ROBOTRAN into:

C24*S8+S24*S56*C8

leading to far more efficient trigonometric computation.

Generation of Recursive Schemes

The formalism governing the generation of the direct or inverse dynamics (Equations (3) and (4)), uses a recursivity principle to compute all the variables along the tree-like structure of the MBS. For instance, the kinematic relation which expresses the absolute angular velocity $\omega^{3}$ of a given body 3 with respect to that of its parent body 2, $\omega^{2}$ is written in a vector form as

(15) $$\omega^{3}=\omega^{2}+\Omega^{23}$$

where $\Omega^{23}$ represents the *relative* angular velocity vector of body 2 with respect to body 3.

Among the three components of this vector (and of all vectors recursively computed in the same manner as vector $\omega^{3}$ in (15)), one surprisingly notices that some of them are squarely useless for the final form of the equations of motion (3) or (4). This can represent up to 30% of the intermediate equations such as some components of the above vector Equation (15): the ROBOTRAN symbolic engine can detect all of them before printing, via the use of a double-linked list containing the full set of recursive equations of type (15), and by tracing their individual utility for the final expected result, such as the mass matrix M(q) or the generalized accelerations $\ddot{q}$.

Constrained MBS: Fully Symbolic Models

Let us rewrite the semi-explicit form (12) in an explicit way in terms of the independent accelerations ${\ddot{u}}_{u}$:

(16) $${\ddot{u}}_{u}=f\left(u_{u},{\dot{u}}_{u},u_{d},{\dot{u}}_{d},{\ddot{u}}_{d},frc,trq\right)$$

**Figure 6:**
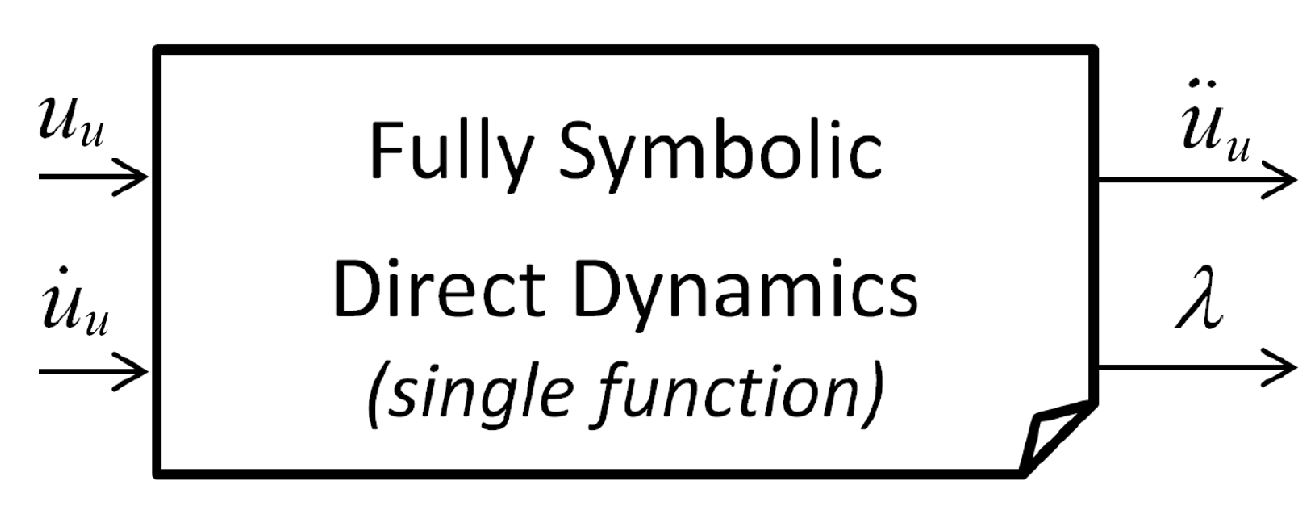
Fully symbolic generation of constrained MBS models.

Thanks to the current ROBOTRAN symbolic engine capabilities, particularly in terms of memory management, it is possible to generate the independent accelerations ${\ddot{u}}_{u}$ according to Equation (16) in a *fully symbolic* manner (Figure 6) and in the form of a unique set of recursive equations of type (15), which successfully compute (in C or Python):

- the constraints and their resolution at position, velocity, and acceleration levels;
- the external forces and torques (interfaced with possible external user constitutive equations);
- the dynamics of the restored tree-like MBS; and
- the reduction to an ODE system and its resolution with respect to the generalized accelerations ${\ddot{u}}_{u}$.

Nowadays, this represents the most efficient symbolic direct dynamics model that ROBOTRAN can provide, making the most of its symbolic engine capabilities. More details on the above symbolic manipulations and tricks underlying the ROBOTRAN process can be found in details in [8].

## 4. Hardware Framework

The dynamic models obtained from the above symbolic multibody approach represent the key ingredient of our haptic device framework. Various competing—or even complementary—approaches can be used to reduce the computational complexity of the model, e.g., via deep learning techniques [31] or to make the most of object-oriented languages [32] and parallel programming [33]. Some results are promising but currently remain limited to rather simple MBS [31]. Regarding parallel computation, let us point out that symbolic generation lends itself perfectly to the vectorization of multibody models [8] and represents a promising avenue of exploitation of GPUs or FPGAs architectures in order to further improve model computation performances.

This section will detail the relationship between the multibody model and the human, that are both *in-the-loop*. In particular, we will describe the coupling between ROBOTRAN and the middleware ROS.

### 4.1. Specifications

First, as the haptic device is designed to be manipulated by a person, the apparatus specifications must meet some requirements of the human body. The human somatosensory system is composed of many sensors present at different levels of perception. According to the authors of [34], the exact contribution of the various mechanoreceptive channels to the formation of haptic perception remains to be established. Moreover, the physical values of perception, that are still the topic of fundamental researches, strongly depend on the application at hand. As regards tactile sensitivity, the latter is hard to characterize in term of frequency sensed [35] and force measurable [36] and needs psychophysical experiments to be able to understand the human tactile perception [37]. Generally speaking, it would appear that an update frequency of 1 kHz is considered as acceptable for human interactions [26,34,35].

In the scope of this paper, only the force feedback is considered, as the physical interface of our devices is exactly the same between the real object and its haptic equivalent, see Section 5.1 and Section 5.2. In other words, the above-mentioned tactile sensitivity is intrinsically representative in our applications.

Second, the hardware design of a haptic device is a bidirectional apparatus that contains both motors and sensors, as it is for the human [38]. The latter may inject energy at some point, while receiving energy from the device at another time [39]. This bidirectionality is the most distinguishing feature of haptic interfaces [40].

The sensor resolution must be sufficient, especially when the movement is fast and small [41]. Of course, the sensors must not influence the device behavior, whose dynamics must satisfy the physics laws such as that of Newton [26].

### 4.2. Human-in-the-Loop Haptics with ROBOTRAN

**Figure 7:**
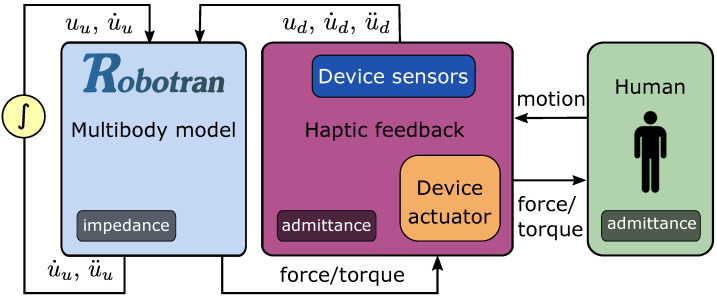
HIL haptic principle with ROBOTRAN. The light blue box, in this case, corresponds to the *Direct Dynamics* of Figure 4.

The multibody approach described in Section 3 can be used to insert a highly dynamic model inside a real-time haptic device, which interacts with a human. Figure 7 shows the relationships between the ROBOTRAN model, the device, and the human body.

In haptics, taking the motion as input and the force as output, and vice versa, are the two possibilities, referred as impedance and admittance approaches in [41]. For the flow diagram shown in Figure 7, the device and the human are considered as an admittance operators, while the model can be assimilated to an impedance one. Other scenarios from Figure 4 are obviously possible.

The haptic device contains sensors that are used to measure the motion and to feed the multibody model. Those sensors are critical as they are in-the-loop located. Any error, noise or non-physic behavior will be reflected in the model and ultimately influence the feedback felt by the human who possesses his own somatosensory system, as discussed in Section 4.1.

Let us note of the importance of integration loop on the left in Figure 7, which must be fast, stable and precise enough. Indeed, the loop involving the human and the haptic device is instantaneous, while the integration loop is unavoidably slower, due to a fixed time-step size and thus a constant computation time.

### 4.3. ROS-ROBOTRAN Coupling

ROS (for “Robot Operating System”) is a open-source software framework widely used in robotics. It contains numerous useful tools and libraries that allow for interactions between individual components, both in terms of software and hardware. ROS allows exploiting these tools in real-time to automate and control an electromechanical device.

Many ROS tools have been created by the ROS community to manipulate sensors or actuators and to publish or receive their data on a network. In addition, ROS offers many other benefits such as the ability to distribute the tasks requiring high processor usage across multiple hardware components. Furthermore, it makes possible to easily modify the system hardware without the need to make major changes in the software.

**Figure 8:**
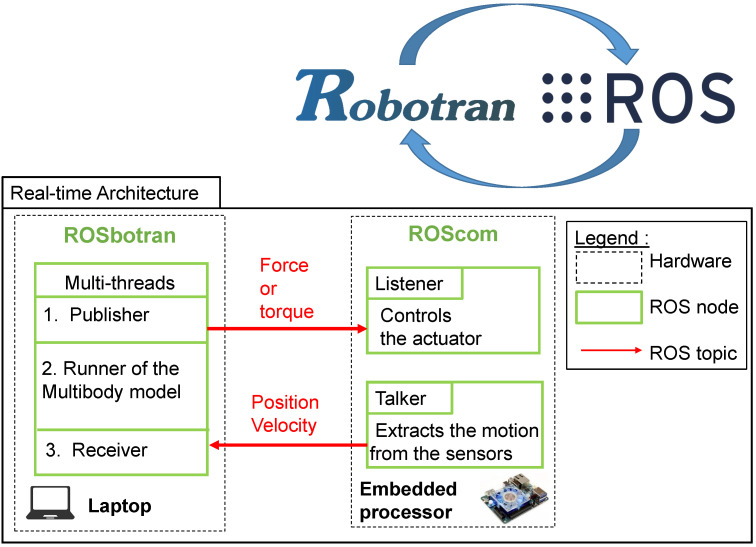
Software architecture with ROS and ROBOTRAN.

The coupling architecture between ROS and ROBOTRAN is presented in Figure 8.

Regarding the software, two packages constitute the basis of the coupling. In ROS, a *package* designates a folder containing one or several executable codes, a configuration file for compiling these, as well as other files useful to ROS. A ROS *node* is an executable that is able to process some computation but also to communicate with other nodes thanks to the ROS *messages* and *topics*.

As far as we are concerned, a ROS-ROBOTRAN package—called **ROSbotran**—has been created, that is able to publish/subscribe to the data of a ROS network. It contains the multibody model running in real-time, which allows to simulate the mechanical system and to forecast, analyze, and control its behavior. Inside this package, for our applications of Section 5, this node uses the multi-threads approach to parallelize the calculations.

Another package—called **ROScom**—has simply a listener–receiver role, containing two nodes: *Listener* and *Talker*. Two *topics* are used to exchange kinematic and dynamic information between the ROSbotran and ROScom packages, see Figure 8. The communication is ensured through the SSH protocol and physically via an ethernet cable.

Regarding the hardware implementation, the ROSbotran package is run on a laptop with a high-frequency *Intel Core i7-9700 CPU 3.6 GHz*. This allows to compute in real-time the multibody models, even if they are complex. An embedded processor—the *Odroid UX4*—hosts the ROScom package, which requires less computing power. This processor ensures the interface with the sensors and actuator via appropriate electronics.

This ROS-ROBOTRAN integration is already operational for several applications (see Section 5) but could be easily generalized to all types of models, sensors and actuators. Let us note that the real-time process considered in this work achieves so-called *soft* requirements. Indeed, the Linux OS used does not meet a *hard* real-time implementation. Future works should implement this approach to specific hardware and adapt the software accordingly.

## 5. Applications

Following the general concepts of Section 4, practical implementations have been designed in our laboratory for rather contrasting applications: a haptic piano key (Section 5.1) and a driving simulator (Section 5.2). Other realizations have also been conceived within the framework of ongoing researches dealing with specific road vehicles.

Several sensors have been utilized in these prototypes, mostly to be ran *in-the-loop*. However, some external sensors presented in this section have also been specifically designed to validate both the multibody model and the corresponding haptic device.

In this section, we take benefits of several sensors to both validate the multibody itself and the ability of the ROS-ROBOTRAN coupling to give a representative feedback to the human.

### 5.1. Haptic Piano Key

**Figure 9:**
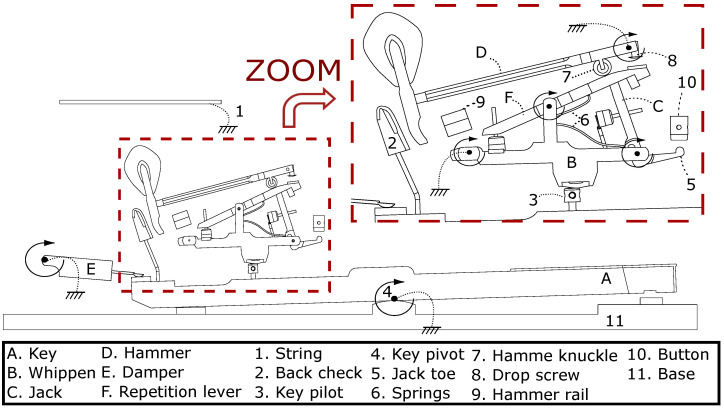
Main components of the grand piano action for the MBS model: mobile bodies (resp. other elements) are indicated by letters (resp. numbers). Circular arrows represent the DOFs (adapted from [43]).

The *touch* of a grand piano mainly results from a dynamic transmission mechanism—called *action*—which propels the hammer up to the string, given the key motion. The multibody model of Figure 9 has been developed at UCLouvain [42].

This model finely represents the action kinematic and dynamic behavior. A first kinematic validation with an external sensor—a high-speed camera—shows that the hammer motion is well caught by the model, a little less well from the back-check capture occurring at the end of the motion, when the key is fully depressed [42].

#### 5.1.1. Previous Realizations

Several researchers have already worked to reproduce the haptic feeling of a piano keyboard thanks to active devices. In this context, the highly dynamic behavior of a piano action could clearly benefit from a multibody approach. Besides, it is difficult to reproduce its effect with only passive elements, as it is done in current digital piano for which manufacturers try to imitate the touch of a real acoustic piano.

In 1993, Gillespie presented the modeling of the piano action dynamics applied within an electromechanical apparatus [28]. The model is derived analytically and contains strong simplifications, which lack some very important dynamic features as the so-called action *escapement, Timmermans2020*. That being said, almost 30 years ago, using the technologies of that time, it was a first trial for a haptic piano key involving a dynamic model.

In 2002, Oboe developed the MIKEY keyboard [44], which allows one to simulate the type of touch of three instruments: the grand piano, the harpsichord, and the Hammond organ. He improved the grand piano action model from Gillespie’s by managing the escapement phase. The considered dynamics still remains simplified compared to the complexity of the piano action. As Gillespie, the experimental prototype uses position sensors and voice coil motors.

Lozada proposed a different approach in 2007 [45], using a magneto-rheological system. He has derived an analytical model of both the physical behavior of the haptic device and the piano action. The key motion is sensed by a contactless photoelectric sensor, and the actuator needs also a force sensing. The final design differs quite significantly from a classic piano key.

With the same purpose to create a haptic piano key, Horvàth proposed in 2014 [46] an experimental fitting of a simplified model with only four constant parameters. He used force and optical sensor to relate the torque acting on the key under different impulses. Unfortunately, no further works has been published by the author to show the practical implementation inside a haptic device.

A broader platform called GENESIS-RT has been introduced by Leonard in 2015 [47] whose goal is to reproduce, among others, the piano action feedback. The piano model is based on a piano-inspired mechanics. The sensors and real-time update rates are quite high, 44 kHz and 4.41 kHz, respectively. No experimental results are shown with the device.

More recently in 2019, Adamou has suggested a two steps approach in [48]. First, an original measurement device has been used to characterize the force feedback according to the key position and velocity. Then, a replication device has been build with an electromagnetic actuator combined with a spring. A laser sensor is placed under the key to measure its position. Again, no validation with the proposed design is proposed.

Those previous attempts have used relatively few sensors, such as optical position sensors or force sensors. The goal was of course to limit the complexity and the cost of such devices. From our perspective, in a purely research context, using multiple sensors of high quality could help in quantifying the haptic feedback quality and prove the approach feasibility. Afterwards, one can imagine to simplify the design complexity for a possible industrial application. For instance, sensors could be integrated in the actuator itself, sensing the position through sensorless methods [49,50,51].

Using the multibody approach described in Section 3, a haptic piano key has been recently developed in our laboratory, with an experimental validation of its force feedback [30]. This implementation is described in the next section. Some improvements brought to the device, as well as further experimental validation are the subject of the subsequent Section 5.1.3, Section 5.1.4 and Section 5.1.5.

#### 5.1.2. Mechatronic Implementation

**Figure 10:**
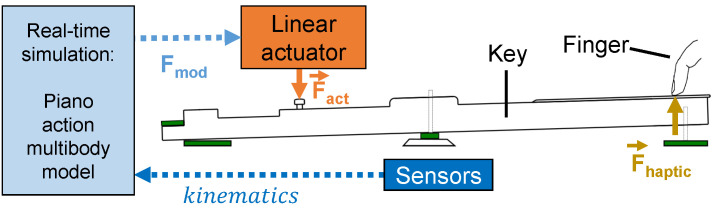
Piano action multibody model force feedback principle, adapted from the work in [30].

For the pianists, the force feedback principle aims at reproducing the same *touch* sensation, i.e., $F_{\mathrm{haptic}}$ in Figure 10. An active device reproduces the force $F_{\mathrm{act}}$ on the key pilot, based on the reference force $F_{\mathrm{mod}}$ computed by the multibody model.

Kinematic sensors measure in real-time the key angular position and velocity, which are inputs of the multibody model, as shown in Figure 7.

**Figure 11:**
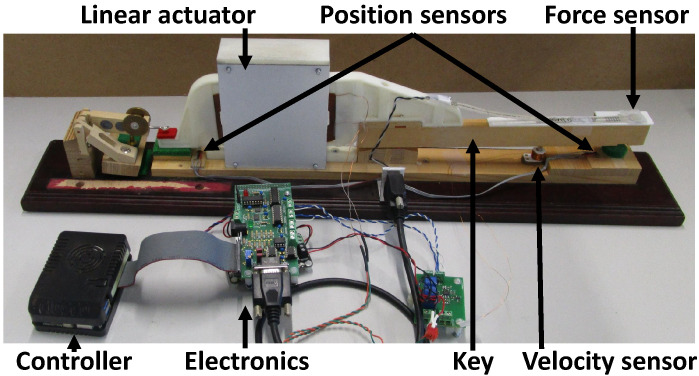
Mechatronic design of one key haptic feedback device, enhanced from the work in [52].

Our prototype (Figure 11) consists of a linear actuator rigidly attached to a piano key. An embedded controller computes the model in real-time (i.e., in less than 1 ms), while the electronic boards drive the actuator accordingly. More details about the mechatronic design are given in [30].

#### 5.1.3. Model Validation

**Figure 12:**
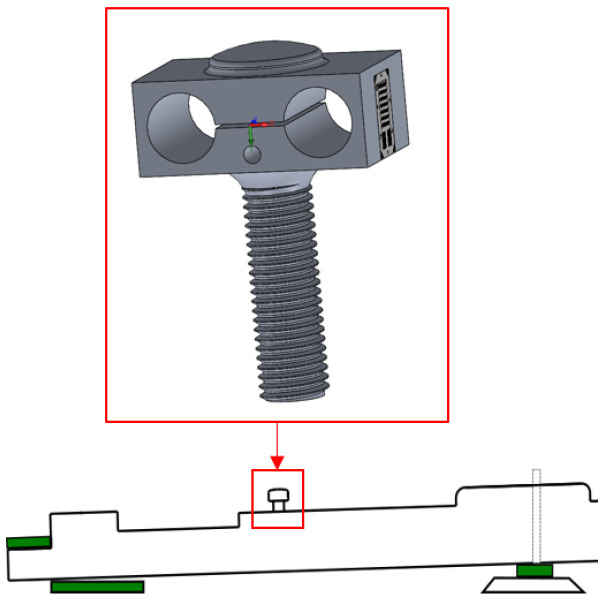
Load cell design.

The model output force $F_{\mathrm{mod}}$ should render the force at the key pilot that would cause a force $F_{\mathrm{haptic}}$ equivalent to that of an acoustic piano keyboard. A custom force sensor has been developed that is based on strain gauges. The sensor sensitivity is 0.765 mV/V, for a measuring range from 0 up to 50 N, with a sampling frequency of 50 kHz, and with a resolution below the milliNewton. The designed load cell of Figure 12 will be inserted instead of the key pilot number 3 in Figure 9 to capture the strain so that the sensor can measure the force applied by the whippen to the key pilot. The latter is exactly the force $F_{\mathrm{act}}$ of Figure 10 that the actuator has to apply in the haptic device. This allows us to validate the output force of the multibody model, see Figure 7.

**Figure 13:**
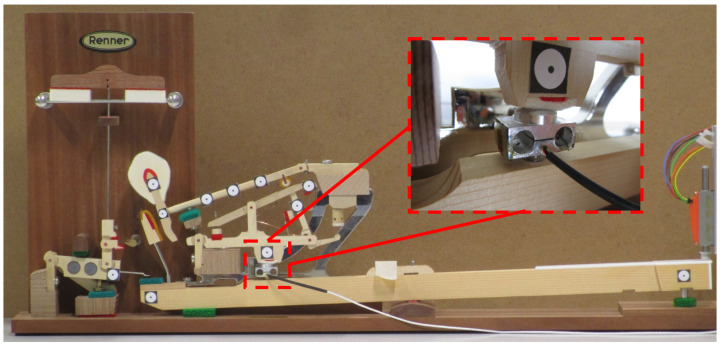
Experimental homemade force sensor set-up.

The experimental sensor is inserted in a real piano action demonstrator, a Renner^®^ action (Figure 13). This homemade binocular element contains four strain gauges glued to the sides, connected through a Wheatstone bridge. This setup allows to precisely measure the normal force between the whippen and the key pilot, without influencing the action dynamic behavior.

The key is actuated by an external actuator shown at the right of Figure 13. A high-speed camera at 1000 fps uses the markers to retrieve the action kinematics. The key motion is then supplied as a model input. This allows to validate the model dynamics, considering the measured motion as the reference, keeping in mind the limitations of the sensing method.

**Figure 14:**
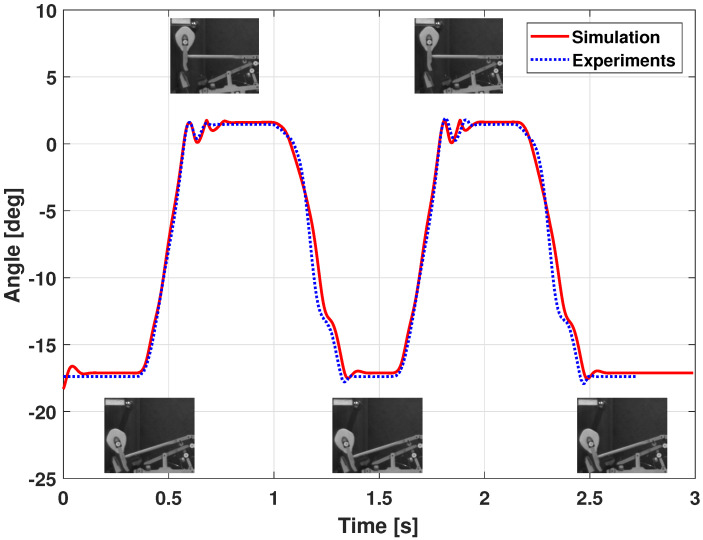
Piano action model dynamic validation: hammer joint position (slow double keystroke).

In Figure 14, a first experiment shows the hammer behavior, compared to the modeling and the measured angle with the high speed camera. Hammer angles are very close in case of this relatively slow double keystroke.

**Figure 15:**
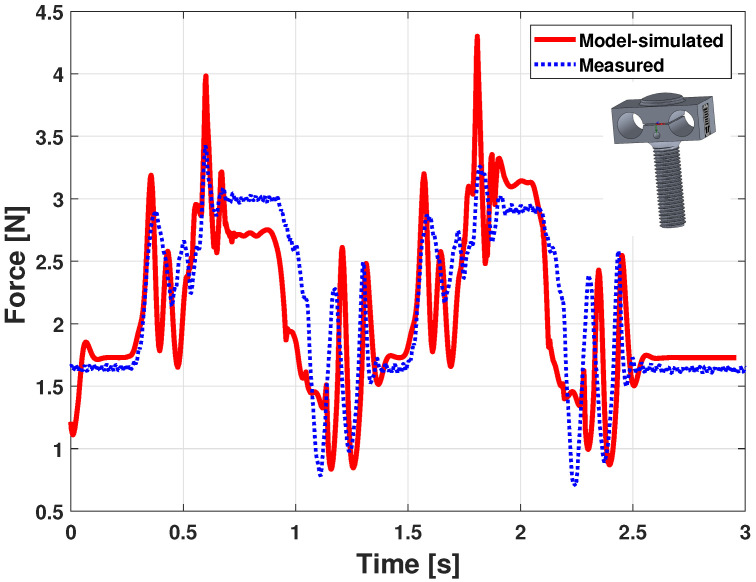
Piano action model dynamic validation: force (slow double keystroke).

Results of the measured force versus the offline model-simulated force are shown in Figure 15. A double blow pattern is visible, the first key strike starting around 0.3 s and the second around 1.5 s. In both cases, the force starts by increasing, with oscillations, until a quasi steady-state value close to 3 N. Then, when the key is released—around 0.9 s and 2.1 s—the action returns to its resting position, with forces peaks due to the hammer rebound, whose mass and inertia dynamic contributions are relatively high compared to other elements.

In Figure 15, the simulation presents more oscillations all along the curve and shows higher force peaks. Apart from that, the measured force is very similar, which is encouraging for the model quality and its ability to finely reproduce the dynamic behavior of a piano action.

**Figure 16:**
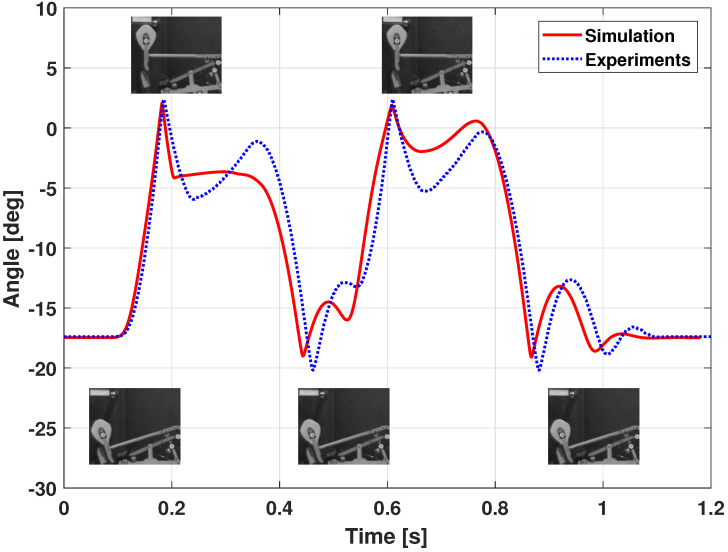
Piano action model dynamic validation: hammer joint position (fast double keystroke).

**Figure 17:**
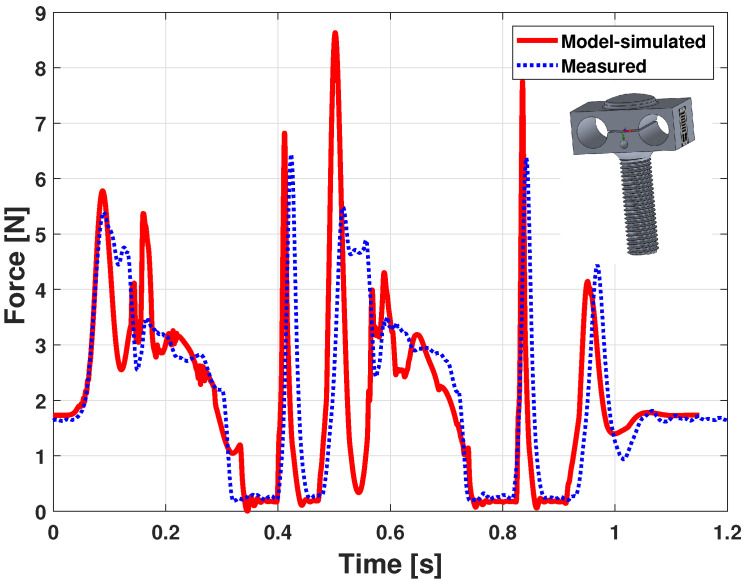
Piano action model dynamic validation: force (fast double keystroke).

A faster double blow is experimented in Figure 16 and Figure 17.

The hammer behavior is not identical between the simulation and the experiments after the hammer–string contact in Figure 16. In fact, the repetition lever acts differently: in experiments, the lever moves the hammer upwards around [0.25;0.35] s and [0.7;0.8] s, while the simulated behavior is different. Indeed, the repetition lever is difficult to tune both in practice and in simulation.

Despite that, the forces of Figure 17 are very similar, apart from the first part of the strokes, around 0.1 s and 0.5 s. Being a fast double blow, those differences are acceptable, and these results show rather good agreements with experiments, given all the remaining model parameter uncertainties.

#### 5.1.4. Real-Time Sensors Validation

The kinematic input supplied to the multibody model, see Figure 7, is of the utmost importance for the quality of the haptic feedback. Noise, oscillations, or error in the input would unavoidably result in a non-realistic force computed by the multibody model.

In the piano key haptic device of Figure 11, two position sensors and a velocity sensor capture the key kinematics to continuously drive the multibody simulation.

The key angle is retrieved through two Hall effect *Allegro A1301* sensors placed in opposition under the key, one at the front and one at the rear, as presented in Figure 11. These two sensors measure respectively the voltages $U_{\mathrm{front}}$ and $U_{\mathrm{rear}}$.

Hall sensors use magnetic field measurements which are not linear versus displacement. However, a chosen combination $U_{\mathrm{combined}}$ between $U_{\mathrm{front}}$ and $U_{\mathrm{rear}}$ allow obtaining experimentally a linear relation between the measurements and the key front height:

(17) $$U_{\mathrm{combined}}=k\cdot \left(U_{\mathrm{front}}^{2}-U_{\mathrm{rear}}^{2}\right)+O$$

where *k* is a proportionality factor determined by calibration and *O* is a fixed offset, also determined by experiments. The $U_{\mathrm{combined}}$ can then be converted to a position measurement and finally to the key angle via simple trigonometry.

The velocity sensor of Figure 11 is an homemade voice coil, with a copper coil fixed on the frame and a moving permanent magnet glued on the key. The motion of the magnet creates a magnetic field variation inside the coil, inducing a varying *back-electromotive* force in the coil. This way, a voltage $U_{\mathrm{coil}}$ is created between the coil wire edges, whose value is directly proportional to the speed of the coil:

(18) $$U_{\mathrm{coil}}=\kappa \;\dot{z}$$

where κ is a proportionality factor found by experiment and linked to the coil characteristics and $\dot{z}$ is the moving magnet vertical velocity. This velocity is considered as purely vertical because the key angle remains small.

**Figure 18:**
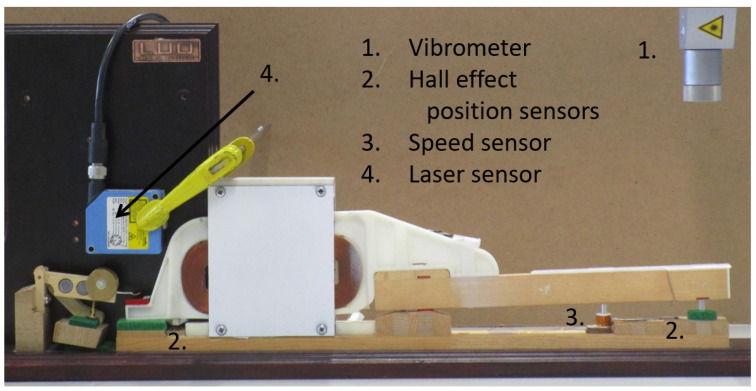
Piano haptic key: kinematics sensing validation.

To validate those position and velocity sensors, an external *Polytec OFV-534* vibrometer is used, as presented in Figure 18. This vibrometer independently measures the position and the velocity with a resolution of 175 μm and 4.4 mm/s in our setup.

**Figure 19:**
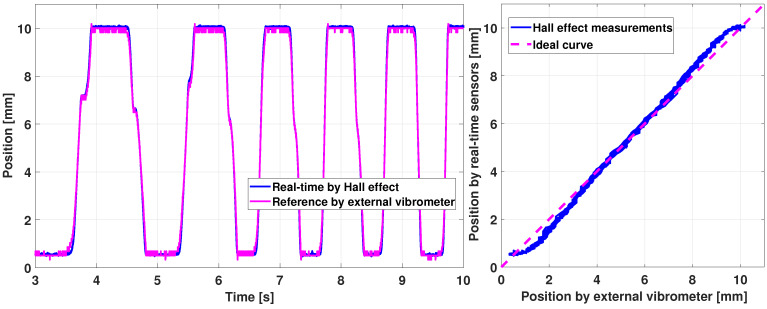
Haptic key validation of position sensors: slow motion.

First, the position estimation is experimented by manually applying up and down movements to the key, as shown in Figure 19.

The Hall effect real-time measured position is close to the reference obtained with the vibrometer. The right graph in Figure 19 shows the relation between measured and reference positions.

**Figure 20:**
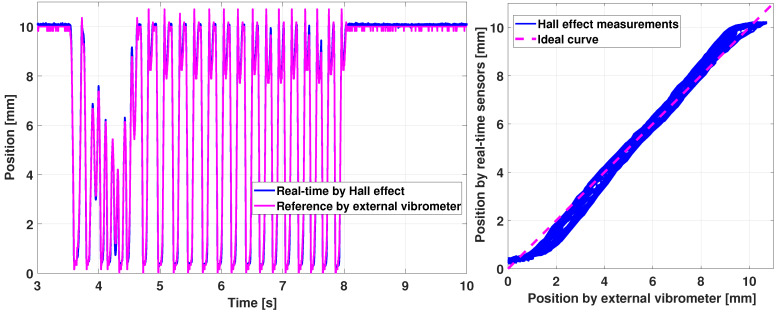
Haptic key validation of position sensors: fast motion.

The experiment of Figure 19 includes a motion at slow frequency, less than 1 Hz. Performing the exact the process at a higher input frequency, around 5 Hz, provides the results shown in Figure 20. The position sensing is still consistent. However, discrepancies appear that cause the Hall effect measurement to have a maximum error of 10% between the estimation and the reality, which could be enhanced, but is sufficient for the considered application.

Furthermore, the combination of (17) allows to maintain the sensor sensitivity, i.e., the slope of the right curves in Figure 19 and Figure 20. Indeed, the relation between the measured position and the real one of Figure 19 has almost a constant slope, except for a small quadratic deviation at the lower and upper position between [0;1] and [9;10] mm, due to the magnetic field quadratic relation with the position. This does not impact the haptic prototype behavior but it can be improved in the future by enhancing the position sensing via a better calibration.

**Figure 21:**
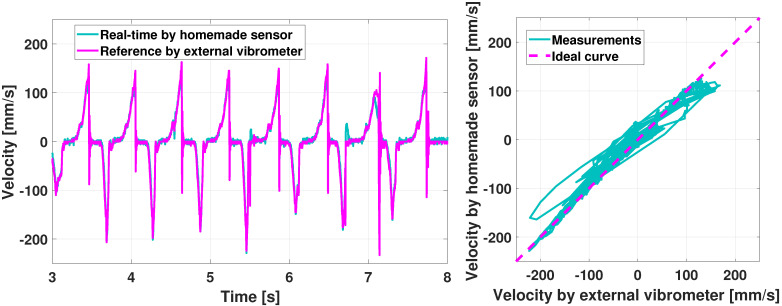
Haptic key validation of the velocity sensor: high amplitude, fast motion.

Regarding the velocity, several up and down motions are again manually applied to the key, and the homemade coil sensor output is compared to the vibrometer measurements in Figure 21.

The homemade sensor captures the velocity amplitude quite well, despite some erroneous values at some points. Indeed, some peaks are present with the homemade but not with the vibrometer, for instance, around 5.5 and 6.7 s in Figure 21, left.

**Figure 22:**
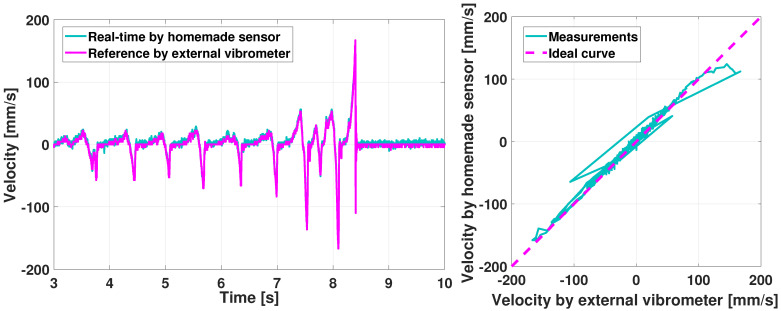
Haptic key validation of the velocity sensor: halfway keystroke.

Figure 22 shows the same experiment for *halfway* keystroke, for which the key is not fully depressed but the action is still able to repeat the note, as foreseen by the so-called *double escapement* grand piano action: this feature is fundamental for pianists. The validation of Figure 22 presents our sensor capability to capture this specific movement.

#### 5.1.5. Haptic Key Results

In [30], the actuator design of our haptic prototype (Figure 11) was exposed and its ability to reproduce the force on the piano key was tested. In the present section, we analyze in further detail the haptic force felt by the pianist in various conditions. For this purpose, we take advantage of the ROS-ROBOTRAN coupling presented above in order to investigate various settings of the piano action like the position of the button height or the mass of the hammer.

**Figure 23:**
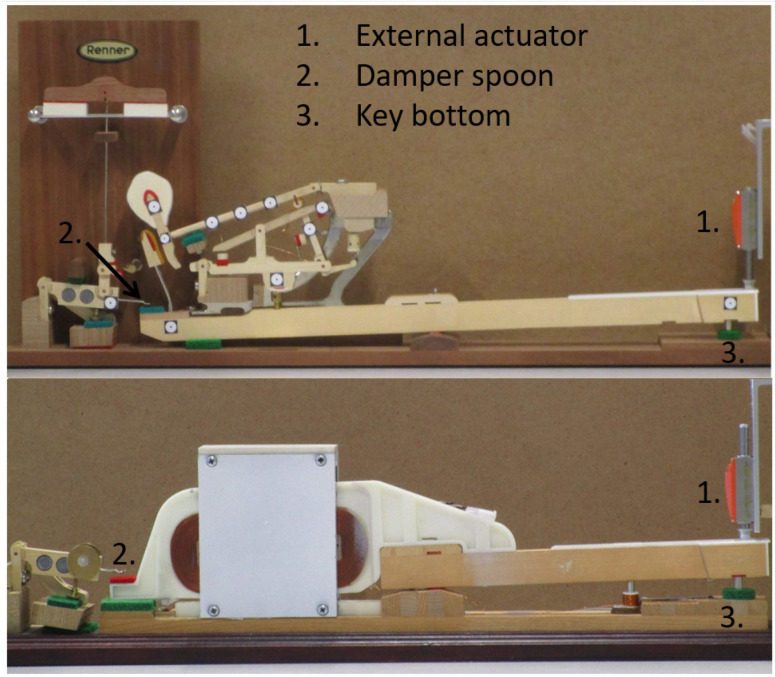
Piano haptic key: measurements of the force felt by the pianist $F_{\mathrm{haptic}}$ through an external linear actuator for the Renner^®^ demonstrator (**up**) and the haptic prototype (**bottom**) [30].

The force felt by the pianist is $F_{\mathrm{haptic}}$ of Figure 10, which depends of course of $F_{\mathrm{mod}}$ and $F_{\mathrm{act}}$ but also on the physical components of the prototype. To characterize $F_{\mathrm{haptic}}$, an external actuator *Faulhaber LM1247* has been used to apply the same position-driven profile that corresponds to one full key dip at 10 mm/s. The $F_{\mathrm{haptic}}$ force can be deduced from the external actuator current measurement on both the action demonstrator and the prototype, see Figure 23.

**Figure 24:**
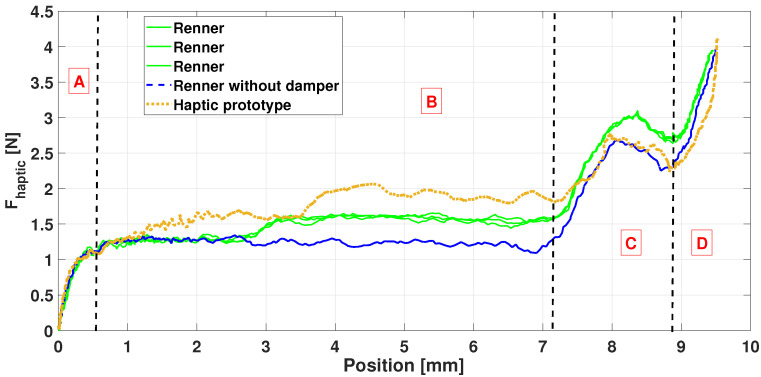
Haptic piano key force validation: prototype comparison with the Renner^®^ demonstrator (results confirming those obtained in [30]).

We first reproduced and checked the test in [30] with the additional sensors presented and validated above. Figure 24 shows $F_{\mathrm{haptic}}$ versus the key vertical position measured at its tip. A key at rest refers to position zero and position 9.5 mm to a fully depressed key, as proposed by [53]. We clearly retrieve the phases A-B-C-D as described in [30].

In addition, similar behaviors can be observed in the profiles of Figure 24, despite some differences due to the physical components that differ between the prototype and the demonstrator. For instance, the blue curve shows the action behavior, without the damper, illustrating its role. The measure on the action demonstrator has been performed three times in a row to highlight the consistency between experiments.

In short, the crucial *escapement* phenomenon occurs during phase C in Figure 24. The difference in the force amplitude between the prototype and the reference may be due to residual inaccuracies in the MBS model.

To illustrate the interest of having a multibody model included in the haptic prototype, variations of the action parameters can be done with the prototype. Their effects on the haptic feedback can be compared with the Renner^®^ demonstrator for which these values have been physically modified.

**Figure 25:**
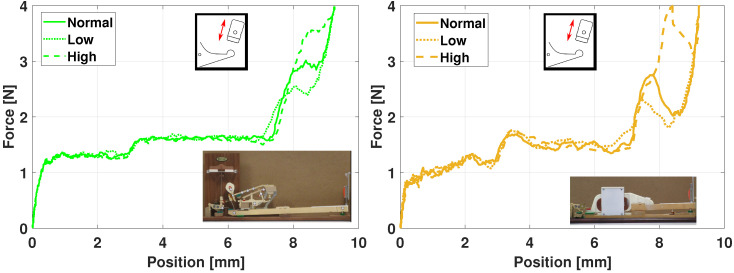
Haptic piano key force validation: variation of the button height, physically on the Renner^®^ demonstrator (**left**) and virtually on the haptic key prototype (**right**).

Figure 25 presents the haptic force for the Renner^®^ (left) and the haptic prototype (right). The parameter is the height of the let-off button [43], see also Figure 9. In the legend, *Normal* means that the setting value is nominal and *Low* (resp. *High*) that the let-off button is lower (resp. higher) than its nominal height, by approximately 0.5 mm.

In Figure 25, the trends are similar between the prototype and the demonstrator: a higher (resp. lower) button causes the escapement phase to have a higher (resp. lower) force value around [8–9] mm. The escapement is even more stressed in the prototype.

**Figure 26:**
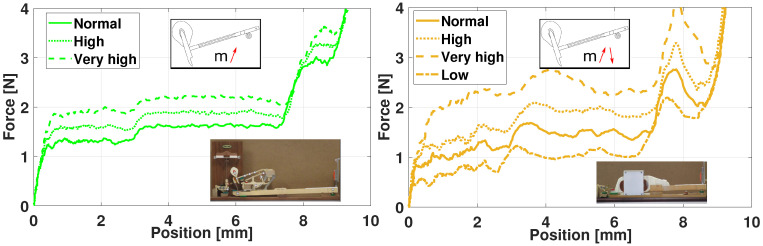
Haptic piano key force validation: variation of the hammer mass, physically on the Renner^®^ demonstrator (**left**) and virtually on the haptic key prototype (**right**).

Figure 26 shows the effects of the hammer mass variation, i.e., meaning that a punctual mass of 0.003 kg has been added once (resp. twice) for the *High* (resp. *Very high*) case.

Again, the impact is similar for the *High* and *Very high* cases, with an increase of the haptic force because the hammer is heavier.

Besides, one advantage of the multibody model is that it can virtually perform many interesting investigations, for instance, the hammer mass can easily be lowered. Doing so in real life would require to manufacture a whole new hammer. This variation is illustrated with the *Low* curve in Figure 26, for the haptic prototype only, resulting with a lower haptic force until the key-bottom contact (phase D in Figure 24).

Despite some discrepancies, the above results (Figure 24, Figure 25 and Figure 26) show that the ROS-ROBOTRAN coupling proposed in this paper allows to develop a haptic key for digital pianos able to reproduce the $F_{\mathrm{haptic}}$ action dynamic force quite faithfully, i.e., the *gold-standard touch* of a grand piano. Moreover, the symbolic multibody modeling approach makes it very easy to modify any physical parameter of the action or even the action itself in the haptic device, to modulate the haptic force accordingly. This feature was actually appreciated by some pianists and piano tuners who were consulted for this haptic keyboard project.

### 5.2. Haptic Driving Simulator

**Figure 27:**
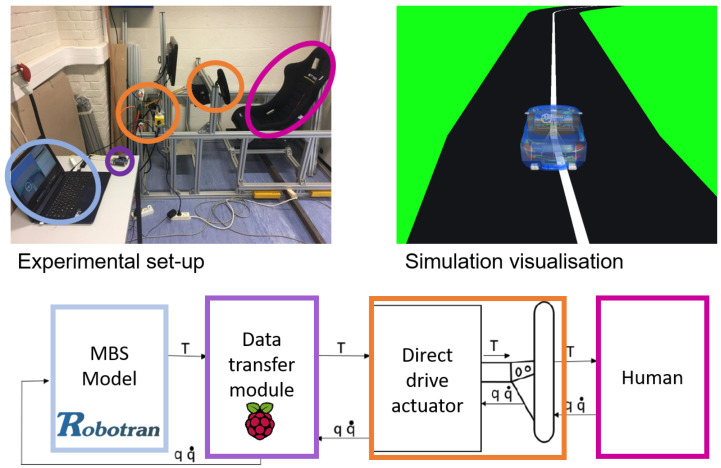
Haptic steering wheel: experimental setup with its real-time visualization and its corresponding block diagram below.

Simulators for vehicle driving are nowadays a common tool and used for many applications [54]. Using a real-time multibody model including all the suitable physical parameters, allows to deal with the highly dynamics effects of a vehicle behavior. To analyze the dynamic performances of a vehicle, other approaches consider for example object-oriented programming of autonomous virtual drivers [32], instead of a real human. A prototype has been built that aims at reproducing the handling torque feedback in the steering wheel. Figure 27 shows the experimental set-up with the block schematics. As in all simulators, the driver has a real-time visualisation of the moving environment on a front screen.

In this haptic demonstrator, mainly developed at UCLouvain for educational purpose, a direct drive actuator acts on the steering wheel rotation. It contains an absolute angular encoder *SinCos Hiperface SKM36 Multiturn* which measures the position *q* and the velocity $\dot{q}$ of the steering wheel. This information is sent through the *data transfer module*—in this case, an embedded processor *Raspberry Pi 3*—to the multibody model of the a full 3D vehicle. Afterwards, the corresponding torque *T*—computed by the inverse dynamics Equation (13)—is applied by the actuator to the human arms.

In the following illustrative experiment, in which a driver handles a virtual vehicle, the situation represents an obstacle avoidance while driving on a straight line at constant speed of 75 km/h. During the simulation, an obstacle suddenly and randomly appears, and the driver needs to avoid it by turning on the left and then coming back on the straight line. Meanwhile, for each of the three simulations, the caster distance of the front suspensions is modified without notifying it to the driver.

**Figure 28:**
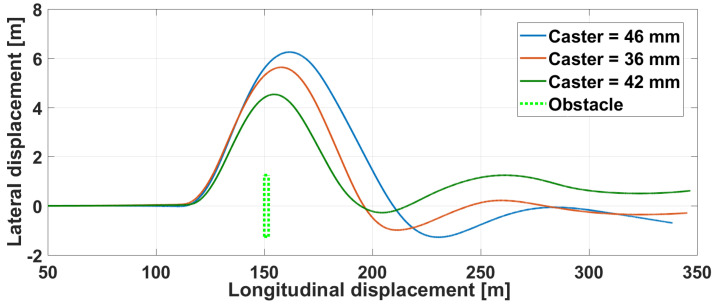
Haptic steering wheel: vehicle displacement on the ground with various caster.

Figure 28 shows the corresponding lateral versus longitudinal vehicle displacements, as well as the obstacle. Without going into a detailed analysis, one can see that the behavior clearly differs depending on the trials and on the setting of the caster.

**Figure 29:**
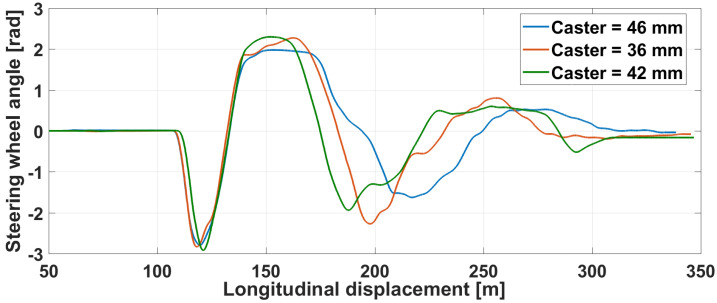
Haptic steering wheel: angle with various caster.

The steering wheel angle, visible in Figure 29, illustrates in a different way the reaction of the driver to the obstacle apparition.

**Figure 30:**
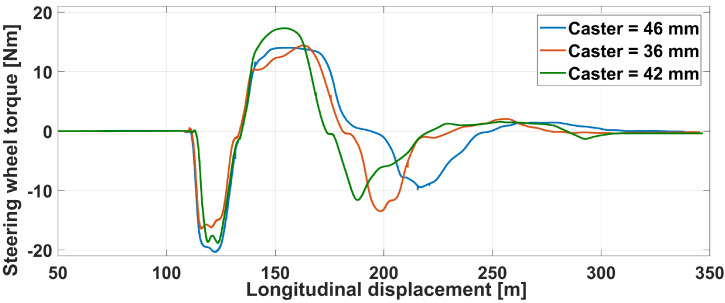
Haptic steering wheel: torque with various caster.

Finally, the torque given as a feedback is shown in Figure 30. Note that this value is taken from the model output, directly in the loop, not measured with an external sensor. For the first negative peak and for a very close angle value, the feedback torque is higher for a higher caster, as expected. After that, the driver counter-steers and tries to stabilize the vehicle towards its initial trajectory.

While a wide range of experiments could be achieved to relate the caster to the driver behavior, this illustrative example clearly highlights the capabilities of a multibody-based haptic steering wheel.

The prototype currently lacks the possibility to control the vehicle velocity. Simulations are done at a constant speed. Current work is ongoing to add *Penny&Giles HLP190* potentiometers to measure the position of the existing brake and accelerator pedals. Adding these sensors would allow to instantaneously adapt the vehicle velocity. Thanks to the ROS-ROBOTRAN coupling described in the previous sections, adding this hardware is quite straightforward in this software environment.

In the future, more experiments can be envisaged with several types of drivers, to analyze their feeling and torque feedback. This way, a wide range of vehicle dynamic parameters can be analyzed, such as the wheel toe-in/toe-out, the tire friction, the roll center height or the anti-roll bar stiffness, among others.

### 5.3. Other Implementations

The two projects presented before constitute the main current applications of the ROS-ROBOTRAN approach. However, other implementations involving a coupling between multibody models with real-time environments have been developed. In this scope, various sensors have been customized and utilized, showing our growing interest in coupling multibody dynamics with sensors for validation or haptic purposes.

**Figure 31:**
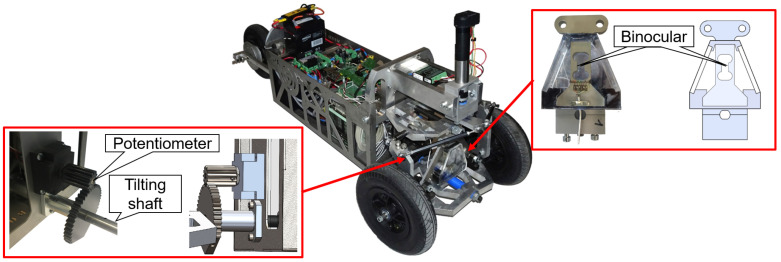
NTV demonstrator and its two main sensors: a rotating potentiometer and a binocular strain gauge for torque measurement.

For instance, during the Ph.D. thesis of Docquier [55] at UCLouvain, a small-scale demonstrator of a Narrow Tilting Vehicles (NTV) has been designed. The demonstrator allows to test embedded controllers thanks to inboard sensors that are used in real-time via ROS to update and analyze the vehicle behavior whose multibody model was built in parallel. Figure 31 presents the prototype and zooms on the two main sensors.

Besides a classical potentiometer connected to the titling shaft to measure the vehicle tilt, a torque sensor based on a binocular strain gauge provides information associated with the actuated tilting degree of freedom of the vehicle. This information is thereafter used for control and validation purposes. Let us note that the vehicle also carries an IMU on board, to combine the tilting value from both IMU and potentiometer with a Kalman filter.

**Figure 32:**
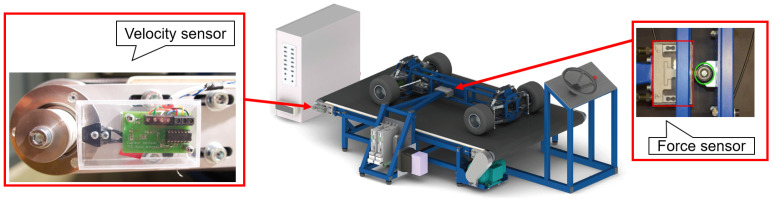
Sensors of the kart bench.

The second project concerns a so-called “kart” bench designed by and for students, to enable them to visualize and understand the road handling behavior of a four-wheel vehicle (Figure 32), and to compare the real system with its multibody model. The kart is laterally held on a conveyor belt and can be human-driven from a remote steering wheel linked to the front suspension via Bowden-type cables. Real-time multibody model can show live the four lateral forces on the tires, for instance, for different types of driving behaviors and/or kart suspension settings.

Apart from a classical optical sensor measuring the conveyor belt velocity, see Figure 32, a longitudinal and transverse force measurement through a homemade binocular load cell with strain gauges is placed between the frame and the moving kart to quantify the lateral force on the kart. It allows to analyze in real-time the effects on the tire-belt interaction forces and also to compare them with those computed live by the multibody model, while modifying suspension settings such as the toe-in/toe-out.

Let us note that this prototype does not run with ROS. Instead, it exploits *Labview* to communicate with the dedicated electronics and to retrieve the necessary sensors information, as for the above-mentioned applications.

## 6. Conclusions and Prospects

The main objective of this work was to highlight that the current multibody modeling formulations had reached a sufficient maturity to be exploited within haptic devices applied to mechanical systems involving high dynamics.

In a first step, we presented a multibody formalism in relative coordinates whose constraint equations are eliminated by a proper reduction of the equations. This formalism lends itself perfectly to its programming via the symbolic approach whose capacity of equation manipulation and simplification allows to produce very compact models, i.e., perfect candidates to real-time computation.

The interaction and coupling of these models with the world of sensors is essential, on the one hand, to validate the models themselves with respect to their underlying physics and, on the other hand, to allow a reliable coupling between the model, the sensors, and the actuators of a haptic device. The ROS platform has been chosen as the meta architecture to ensure these couplings successfully: two applications illustrate our developments. The first one, presented in detail, concerns the development of a haptic piano key and demonstrates the capabilities of the approach for a very high dynamic system. The second one refers to a driver simulator under development in our laboratory, whose interest—above all pedagogical—is to show the appeal of multibody models for this kind of application. In particular, it allows students to observe the impact of different parameters on the system dynamics, as taught in the academic courses but through multibody equations that can be a little bit abstruse for some of them.

In terms of perspectives, it would be interesting to continue the investigations on the model side, in particular through the fine-grain parallelization of the symbolic equations. Preliminary tests in ROBOTRAN have indeed shown that recursive equations can be reorganized in very few sequential vectorial steps: this asset could be exploited in order to further reduce the computation time of models to be embedded in devices requiring real-time computation.

Besides, we think that the potentialities of coupling real-time multibody dynamics with sensors and actuators within a middleware platform (e.g., ROS) are very promising for the future. It will enable to develop new concepts of haptic systems that are generic, user-friendly, and efficient. In particular, we see a real pedagogical interest in the use of such an architecture for the fields of multibody modeling, sensor implementation and mechatronic design of haptic systems with a very pronounced dynamic character, such as those presented in the present work.

## Data Availability

Not applicable.

## Abbreviations

The following abbreviations are used in this manuscript:

CPU	Central Processing Unit
DAE	Differential-Algebraic Equation
DOF	Degree of Freedom
FPGA	Field Programmable Gate Area
GPU	Graphics Processing Unit
HIL	Human-in-the-loop
IMU	Inertial Measurement Unit
MBS	Multibody Systems
NTV	Narrow Tilting Vehicles
ODE	Ordinary Differential Equation
OS	Operating System
RAM	Random Access Memory
ROS	Robot Operation System
SSH	Secure Shell
YARP	Yet Another Robot Platform
